# Methane-Dependent Extracellular Electron Transfer at the Bioanode by the Anaerobic Archaeal Methanotroph “*Candidatus* Methanoperedens”

**DOI:** 10.3389/fmicb.2022.820989

**Published:** 2022-04-12

**Authors:** Heleen T. Ouboter, Tom Berben, Stefanie Berger, Mike S. M. Jetten, Tom Sleutels, Annemiek Ter Heijne, Cornelia U. Welte

**Affiliations:** ^1^Institute for Water and Wetland Research, Department of Microbiology, Radboud University, Nijmegen, Netherlands; ^2^Wetsus, European Center of Excellence for Sustainable Water Technology, Leeuwarden, Netherlands; ^3^Faculty of Science and Engineering, University of Groningen, Groningen, Netherlands; ^4^Environmental Technology, Wageningen University, Wageningen, Netherlands

**Keywords:** ANME-2d, extracellular electron transfer, anaerobic methane oxidation, cytochromes, biofilms, bioelectrochemistry

## Abstract

Anaerobic methanotrophic (ANME) archaea have recently been reported to be capable of using insoluble extracellular electron acceptors *via* extracellular electron transfer (EET). In this study, we investigated EET by a microbial community dominated by “*Candidatus* Methanoperedens” archaea at the anode of a bioelectrochemical system (BES) poised at 0 V vs. standard hydrogen electrode (SHE), in this way measuring current as a direct proxy of EET by this community. After inoculation of the BES, the maximum current density was 274 mA m^–2^ (stable current up to 39 mA m^–2^). Concomitant conversion of ^13^CH_4_ into ^13^CO_2_ demonstrated that current production was methane-dependent, with 38% of the current attributed directly to methane supply. Based on the current production and methane uptake in a closed system, the Coulombic efficiency was about 17%. Polarization curves demonstrated that the current was limited by microbial activity at potentials above 0 V. The metatranscriptome of the inoculum was mined for the expression of *c*-type cytochromes potentially used for EET, which led to the identification of several multiheme *c*-type cytochrome-encoding genes among the most abundant transcripts in “*Ca*. Methanoperedens.” Our study provides strong indications of EET in ANME archaea and describes a system in which ANME-mediated EET can be investigated under laboratory conditions, which provides new research opportunities for mechanistic studies and possibly the generation of axenic ANME cultures.

## Introduction

Methane (CH_4_) is the most important hydrocarbon in the atmosphere and a potent greenhouse gas (Myhre and Drew, 2013; Dean et al., 2018). The bulk of biogenic methane is produced in oxygen-limited ecosystems such as peat bogs, wetlands, and swamps by methanogenic archaea (Solomon et al., 2007; Van Groenigen et al., 2011; Myhre and Drew, 2013). The majority of the produced methane is, in turn, biologically oxidized by aerobic and anaerobic methanotrophs before it reaches the atmosphere (Dean et al., 2018). Aerobic methanotrophs require oxygen for methane activation by methane monooxygenase, and as the terminal electron acceptor of their respiratory chain. The aerobic methane metabolism to CO_2_ proceeds *via* methanol, formaldehyde, and formate (Knief, 2015; Schmitz et al., 2021). Anaerobic methanotrophic (ANME) archaea are related to methanogenic archaea at the Methanomicrobia class level and are able to activate methane *via* the methyl-coenzyme M reductase (MCR) enzyme complex. ANME archaea can be divided into subgroups, ANME-1, −2abcd, and −3. ANME-2 have recently been reported to use insoluble extracellular metals and metalloids or syntrophic bacteria as the electron acceptors *via* a mechanism named extracellular electron transfer (EET) (McGlynn et al., 2015; Cai et al., 2018; Leu et al., 2020a). Anaerobic oxidation of methane (AOM) was first described in consortia containing ANME-1, −2abc, and −3 archaea and sulfate-reducing bacteria (SRB) in marine sediments (Reeburgh and Heggk, 1977; Hoehler et al., 1994; Hinrichs et al., 1999; Boetius et al., 2000; Orphan et al., 2002). Direct interspecies electron transfer (DIET) has been proposed to be the EET mechanism used by these microorganisms (McGlynn et al., 2015). The artificial electron acceptor anthraquinone-2,6-disulfonate (AQDS) successfully uncoupled ANME-2ac from their syntrophic partners (Scheller et al., 2016). For ANME-1, the syntrophic partners could not be uncoupled using molecular intermediates such as H_2_ and formate. Instead, a combination of pili from SRB and cytochromes from both microorganisms have been proposed to directly connect ANME-1 to SRB (Wegener et al., 2015). More specifically, a multiheme *c*-type cytochrome (MHC)–surface (S)-layer fusion protein is a potentially important protein in the DIET mechanism of the ANMEs (McGlynn et al., 2015). Large MHCs are encoded in the genomes of ANME-2abd (Haroon et al., 2013; Arshad et al., 2015; Berger et al., 2017). Large MHCs are mostly found in microorganisms that conduct EET such as *Geobacter*, *Shewanella*, or anammox bacteria (Edwards et al., 2020; Shaw et al., 2020), which indicates that MHCs in ANMEs may be involved in their EET mechanism. Comparative genome studies have reported a large number of MHCs in ANME (Kletzin et al., 2015; McGlynn et al., 2015; Leu et al., 2020b).

“*Candidatus* (*Ca*.) Methanoperedens,” also known as ANME-2d, was first described in 2006 (Raghoebarsing et al., 2006). Experimental evidence was obtained that these archaea were able to oxidize methane with concomitant nitrate reduction (Haroon et al., 2013). “*Ca*. Methanoperedens” have also been detected in environments without nitrate but with alternative electron acceptors such as metal-oxides, arsenate, and selenate (Beal et al., 2009; Lu et al., 2016; Weber et al., 2017; Luo et al., 2018; Shi et al., 2020). Several studies reported AOM concomitant with the reduction of an extracellular electron acceptor in complex mixed cultures with “*Ca*. Methanoperedens” as the dominant microorganism, which implies EET to solid minerals instead of interaction with a syntrophic partner (Ettwig et al., 2016; Bai et al., 2019; Zhang et al., 2019). Further experiments have been conducted with electron shuttles AQDS and AQS, with biochar as the conductive material, humic acids, and ultimately with the solid minerals ferrihydrite and birnessite, all providing strong indications for EET by ANME-2d (Ettwig et al., 2016; Cai et al., 2018; Bai et al., 2019; Zhang et al., 2019; Leu et al., 2020a; Valenzuela et al., 2020). Interestingly, in several of these studies, the electroactive heterotrophic microorganism *Geobacter* was enriched during the experiments, which suggests that either decaying microbial biomass functioned as a substrate or that organic soluble intermediates may have been produced by “*Ca.* Methanoperedens” which were in turn used as a substrate by *Geobacter*. Long-term enrichments have also been performed with “*Ca*. Methanoperedens” and extracellular electron acceptors such as ferrihydrite (Cai et al., 2018) and birnessite (Leu et al., 2020a). These studies provided valuable insights into the physiology of “*Ca*. Methanoperedens” and its potential to perform EET showing the expression of several MHCs while performing AOM with Fe(III) or Mn(IV).

In the previous preliminary research, “*Ca*. Methanoperedens” has been grown in microbial fuel cells (MFCs) with the aim to decouple “*Ca*. Methanoperedens” from the possible syntrophic partners. Ding et al. (2017) incubated “*Ca*. Methanoperedens” in an MFC, where it indeed colonized the electrode surface and circa 25 mV was produced. However, *Geobacter* and *Ignavibacterium* spp., which are well-known electroactive bacteria, enriched at the bioanode too. It is therefore difficult to attribute the generated current in this MFC (Ding et al., 2017) to direct EET by “*Ca*. Methanoperedens.” In another study “*Ca*. Methanoperedens” was grown in a BES in which the potential of the anode was poised at 0.7 V vs. standard hydrogen electrode (SHE) (Zhang et al., 2020) and the soluble redox mediator ferricyanide was added. Without this mediator, no current was generated. Furthermore, higher methane partial pressures seemed to increase the current, which demonstrated that the (at least part of the) current was dependent on methane availability. Surprisingly, in this particular study (Zhang et al., 2020), “*Ca*. Methanoperedens” decreased from 32.4% relative abundance in the inoculum to 25.7% relative abundance in the anode biofilm, which corresponds to a 22% decrease in 255 days. Instead, *Geobacter* emerged at the anode (2.4% relative abundance), similar to what was found by Bai et al. (2019), Ding et al. (2017), and Zhang et al. (2020), while *Geobacter* had been below the detection limit in the inoculum. We previously investigated a “*Ca.* Methanoperedens” dominated culture in a BES poised at + 0.40 V vs. SHE (Berger et al., 2021). Also, in this study, other electroactive microorganisms that emerged in the biofilm suggest that not methane but alternative carbon sources such as acetate were the driving force for current production.

There are still many unanswered questions on whether “*Ca.* Methanoperedens” is capable of EET and, if so, what the underlying mechanism is. The high abundance of *Geobacter* and other electroactive bacteria and the lack of a clear methane dependency need to be addressed. In this study, we therefore investigated the biological current production by a mixed culture dominated by “*Ca*. Methanoperedens” in an anoxic BES with methane as the only electron donor, and an anode poised at 0.0 V vs. SHE. We demonstrate the conversion of (labeled) methane into CO_2_ with concomitant current production and “*Ca*. Methanoperedens” as the only known methanotroph present at the anode, in three independent experiments. We show that this current is only produced in the presence of methane. The analyses of the microbial community composition showed that “*Ca.* Methanoperedens” was still the dominant microorganism at the anode while no other microorganisms emerged here. Metatranscriptome analysis of the inoculum revealed the expression of genes encoding for multiheme protein complexes. This work provides a strong indication for EET by “*Ca.* Methanoperedens” and opens new research directions for EET research in ANME archaea.

## Experimental Procedures

### Enrichment Culture—Inoculum

For the enrichment of “*Candidatus* Methanoperedens sp.,” an anaerobic 5 L sequencing fed-batch reactor was used as described by Vaksmaa et al. (2017) where the FeSO_4_⋅7H_2_O solution was replaced by an iron solution consisting of FeCl_3_⋅6H_2_O (32.4 g/liter) and nitrilotriacetic acid (NTA; 96 g/liter) from which 3 ml was added per 10-L medium. The bioreactor was exposed to nitrate-limiting conditions and continuously operated since March 2014. The bioelectrochemistry experiments were performed between March and June 2020.

### Bioelectrochemical System Operation

**FIGURE 1:**
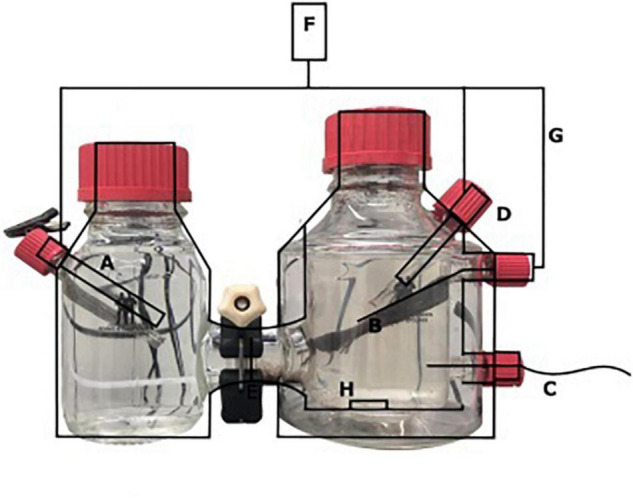
Schematic overview of the BES with **(A)** stainless steel cathode, **(B)** carbon cloth anode (7.2 × 2.5 cm), **(C)** Gas-in CH_4_/CO_2_ 95%/5%, N_2_ > 99% **(D)** Ag/AgCl reference electrode, **(E)** nafion cation exchange membrane, **(F)** potentiostat, **(G)** platinum wire, and **(H)** stirring bar.

A two-chamber bioelectrochemical system (BES) was used that consisted of a jacketed single bottle (anode chamber), which was connected to another single bottle functioning as the cathode chamber (Figure 1) (Adams and Chittenden Scientific Glass, Berkeley, CA, United States). These two chambers were connected *via* a Nafion ion exchange membrane with a thickness of 0.051 mm (Sigma Aldrich, St. Louis, MO, United States). The anode [carbon cloth, (Fuel Cell Earth, Woburn, MA, United States)], the cathode [stainless steel mesh, (Goodfellow, Huntingdon, United Kingdom)], and a 3 M Ag/AgCl reference electrode (+200 mV vs. SHE; Prosense, Oosterhout, Netherlands) were connected to a MultiEmStat3 potentiostat (PalmSense, Houten, Netherlands). The anode and cathode were connected *via* a platinum wire (Goodfellow, Huntingdon, United Kingdom). A potential of 0.0 V vs. SHE was applied to the anode, for which the surface area was 7.2 cm × 2.5 cm. The cathode chamber contained a phosphate buffer (0.15 mM, pH 7.5). The anolyte solution contained per liter, 0.1 g CaCl_2_⋅2H_2_O, 0.07 g MgSO_4_⋅7H_2_O, and 0.05 g KH_2_PO_4_ and was autoclaved prior to the addition of 10 mmol L^–1^ HEPES, 2 ml L^–1^ trace elements (Vaksmaa et al., 2017), and 0.1 ml L^–1^ vitamin solution (Vaksmaa et al., 2017) with a final pH of 7.25 and at room temperature. The anolyte and catholyte were sparged with N_2_ until no oxygen (<10 ppm) was measured in the BES using a Fibox 4 oxygen meter (Presens, Regensburg, Germany). Before inoculation, the current was measured abiotically and was always 0 μA ± 1 μA. The anode of the BES was inoculated with 0.24 g biomass (dry weight) under strictly oxygen-free conditions (<10 ppm) in an anaerobic chamber. Before inoculation, nitrate was measured in the sample and was undetectable using nitrate/nitrite colorimetric test strips (detection limit 10 mg L**^–^**^1^) (MQuant™, Merck, Darmstadt, Germany). The biomass was washed two times with medium before the inoculation to make sure that no lyzed cells or other residues from the bioreactor were transferred to the BES. The anode chamber was continuously sparged with CH_4_/CO_2_ 95%/5% at 15 ml min**^–^**^1^ and N_2_ > 99% at 1.5 ml/min**^–^**^1^. The current was monitored using MultiTrace software (PalmSense, Houten, Netherlands) in chronoamperometric detection mode with measurements taken every 60 s.

Three independent batch experiments were performed (Supplementary Figure 1). After each potential controlled current generation, each batch experiment was used for additional tests. For all three batch experiments, the potential was poised at 0.0 V vs. SHE. In the first experiment (experiment A), 18 h after the start of the experiment, the gas flow was stopped, and 30 ml ^13^CH_4_ (>99%) was added to the anode chamber, which resulted in a pressure of 1.24 bar in the anode. ^13^CO_2_ and ^12^CO_2_ were measured in the headspace over the course of the experiment using gas chromotography-mass spectrometry (GC-MS) (Agilent 5975 inert MSD, Agilent, United States). These measurements were used to calculate ^13^CO_2_/(^13^CO_2_ + ^12^CO_2_) in the headspace over the course of the experiment. Total CH_4_ was measured using gas chromotography (Hewlett Packard 5890, Palo Alto, CA, United States) to follow the consumption of methane. In the second experiment (experiment B), the gas inflow of CH_4_/CO_2_ 95%/5% was stopped and replaced by Argon/CO_2_ 95%/5% to assess the methane dependency of the current production (in duplicate). After the methane was resumed for a while, the CH_4_/CO_2_ gas circulation was stopped again and 30 ml methane was added to create an overpressure. In the third experiment (experiment C), polarization curves were measured at five consecutive days, over different potentials ranging from −0.35 to 0.5 V vs. SHE, taking steps of 0.05 V. For every potential, the current was measured for 5 min.

### Reference Batch Experiment With Nitrate as Electron Acceptor

A batch experiment was performed in a 120-ml anoxic bottle to compare the methane consumption rate of the microbial community using nitrate as the electron acceptor with the rates in the BES. Totally, three bottles were inoculated with 49 mg biomass (dry weight) and a final volume of 30 ml. The same medium was used, and 0.6 mM NaNO_3_ were added at the start of the experiment. Every time the NaNO_3_ was consumed, it was replenished. Methane was measured in the headspace at different time points.

### DNA Sequencing and Metagenome Analysis of the Microbial Community

For experiment A, DNA extractions from the inoculum (*n* = 1) and the biofilm (*n* = 1) were performed using the Powersoil DNeasy kit (Qiagen, Hilden, Germany) according to the manufacturer’s instructions, with the addition of a 10-min bead beating step at 50 s**^–^**^1^ using a TissueLyzer (Qiagen, Hilden, Germany). The library was prepared using the TruSeq DNA PCR-Free Kit (Illumina, San Diego, CA, United States) and sequenced using Illumina NovaSeq 6000, with 150-bp paired-end reads (Macrogen, Seoul, South Korea). The quality of the reads was assessed using FastQC (Babraham Bioinformatics, Babraham institute, Cambridge, United Kingdom), and the reads were trimmed using BBDuk from BBTools (DOE Joint Genome Institute, Walnut Creek, CA, United States), using a minimum read length of 75 bp, and co-assembled into contigs using MEGAHIT (Li et al., 2015). The filtered reads were mapped to the assembled contigs using BBmap (Bushnell, 2014) and sorted using SAMtools (Li et al., 2009). Contigs were binned using MaxBin 2.0 (Wu et al., 2016), MetaBAT (Kang et al., 2015), CONCOCT (Alneberg et al., 2014), and BinSanity (Graham et al., 2017), and binning results were aggregated using DAS tool (Sieber et al., 2018) and refined using anvi’o (Eren et al., 2015). The genome taxonomy database toolkit (GTDB-tk) was used to classify the metagenome-assembled genomes (MAGs). In this process, genomes are classified based on a combination of its placement in the GTDB reference tree, for which marker genes are used, the relative evolutionary divergence (RED) and the average nucleotide identity (ANI) (Chaumeil et al., 2020). Gene calling and annotation were performed using an in-house pipeline (Picone et al., 2020).

The relative abundance of the microorganisms in the microbial community in the inoculum and biofilm at the anode was assessed by assigning sequencing reads to taxonomic ranks using Kraken2 (Wood et al., 2019) at the nucleotide level and Kaiju (Menzel et al., 2016) at the amino acid level. Both outputs were merged with the last common ancestor option, using a script provided in the Kaiju distribution, and the relative abundance was finally estimated based on the classification of the reads using Kaiju. The ANI was calculated using JSpeciesWS^1^ and a genome-to-genome comparison was made based on the digital DNA-DNA hybridization (DDH) using the genome-to-genome distance calculator from dsmz (Meier-Kolthoff et al., 2013). Signal peptides were identified using SignalP 5.0 (Juan et al., 2019), and S-layer domains of the MHCs were identified using the NCBI Conserved Domain scanner^2^.

The metagenome was mined for potential MHCs. The protein fasta file generated by the in-house pipeline for the different bins was mined for sequences that contained at least three occurrences of the C-X-X-C-H heme c-binding motif.

The raw reads are deposited under PRJNA712948 in NCBI, the annotated MAGs are deposited under the accession number PRJEB43626 .

### RNA Sequencing and Metatranscriptome Analysis of the Inoculum

Samples from the inoculum (technical replicates, *n* = 3) were used for metatranscriptomic analysis. The electrode was washed with medium to obtain samples from the electrode, and unfortunately the RNA extractions were unsuccessful due to the small sample size. RNA extractions were performed using the RiboPure-Bacteria Kit according to the manufacturer’s instructions (Thermo Fisher Scientific, Waltham, MA, United States), with the addition of a 15-min bead beating step at 50 s**^–^**^1^ using a TissueLyzer (Qiagen, Hilden, Germany). The extracted RNA was treated with DNase also using the RiboPure-Bacteria Kit according to the manufacturer’s instructions. Quantity and quality of the RNA were assessed with an Agilent 2100 Bioanalyzer. The transcriptome libraries were constructed using the TruSeq^®^ Stranded mRNA Library Prep protocol (Illumina, San Diego, CA, United States) according to the manufacturer’s instructions. Total RNA was used for library preparation, and obtained libraries were checked qualitatively and quantitatively as described above. Pooled libraries were sequenced using the Illumina MiSeq sequence machine (Illumina, San Diego, CA, United States). For sequencing, the 150-bp sequence chemistry was performed using the MiSeq Reagent Kit v3 (Illumina, San Diego, CA, United States) according the manufacturer’s protocol in one direction. SortMeRna (Kopylova and Noe, 2012) was used to filter out rRNA reads by filtering the reads that mapped to the SILVA database. Transcript abundance was estimated by pseudoalignment using Kallisto v0.46.1 (Bray et al., 2016) and expressed as transcripts per million (TPM). The RNA-seq data are deposited under the accession number PRJNA712948 in NCBI.

## Results

### Microbial Community Composition of the Electroactive Community

**FIGURE 2:**
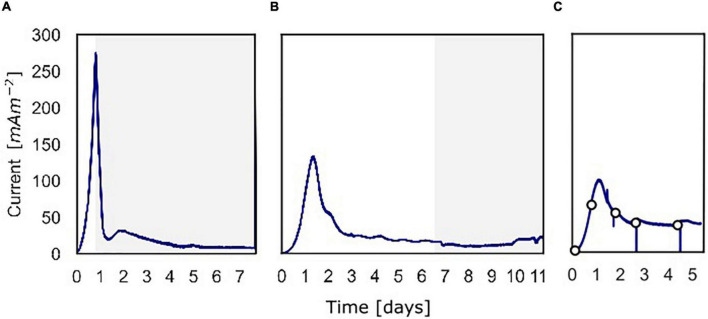
The current density produced over time over the course of the three batch experiments. **(A)** Current density for the experiment in which ^13^C-methane was used to follow the activity of “*Ca.* Methanoperedens,” the part in which ^13^C-methane was added and followed is shown in gray. **(B)** Current density for the experiment in which methane availability was varied to assess methane dependency of the current. The gray part shows when the experiment was performed shown in Figure 5. **(C)** Current density for the experiment in which at different time points a polarization curve was used to assess dependency of methane oxidation on the potential.

**FIGURE 3:**
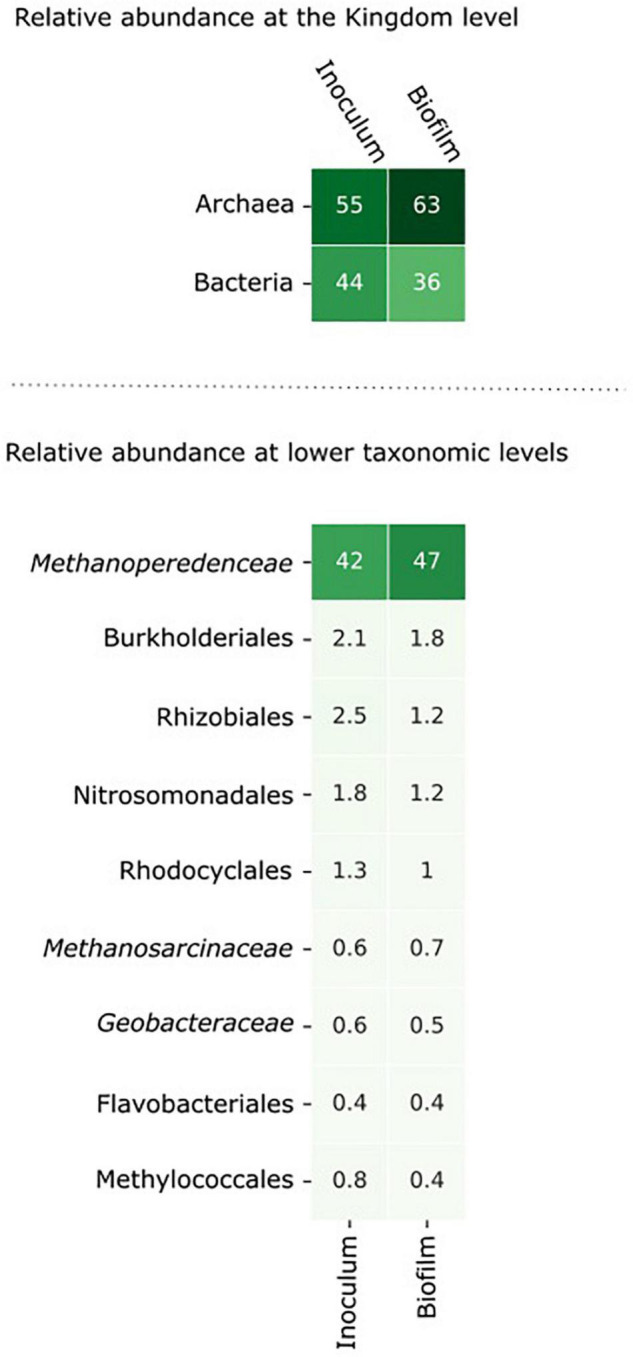
The microbial community composition in the inoculum and in the biofilm at the anode after 8 days of incubation. The relative abundance of the microorganisms was assessed by assigning metagenomic sequencing reads to taxonomic ranks using Kraken2 (Wood et al., 2019) at the nucleotide level and Kaiju (Menzel et al., 2016) at the amino acid level. Microorganisms with an abundance > 0.5% in either the inoculum or the biofilm are shown in the figure.

**FIGURE 4:**
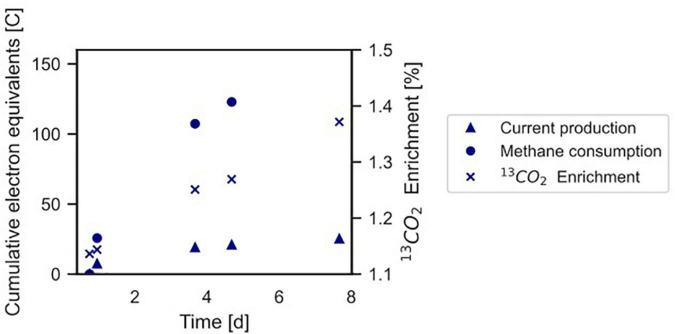
Conversion of ^13^CH_4_ to ^13^CO_2_ by the microbial community in the BES indicating anaerobic methanotrophy. The Coulombic efficiency in this experiment was calculated to be 17.3%. Profiles of methane and current are expressed as cumulative electron equivalents at the primary y-axis, ^13^CO_2_ enrichment (% ^13^CO_2_/Total CO_2_) is shown at the secondary y-axis. The graph refers to experiment A. The area of the electrode is 0.0018 m^2^. •, methane consumption; Δ current production; x ^13^CO_2_ enrichment.

The microbial community composition of both the inoculum and the anode biofilm was analyzed (8 days after the start of the experiment A; Figures 2A, 3, 4 and Supplementary Table 1) using metagenomics. Due to the presence of multiple 16S rRNA genes in some of the microorganisms present in the experiment, we chose to refrain from a quantification method only relying on the analysis of this marker gene. Instead, a more unbiased method for relative abundance estimation was chosen, which makes use of all metagenomic sequencing reads. The relative abundance of microorganisms was estimated directly from the raw reads using a combination of the tools Kraken2 and Kaiju (Menzel et al., 2016; Wood et al., 2019).

**FIGURE 5:**
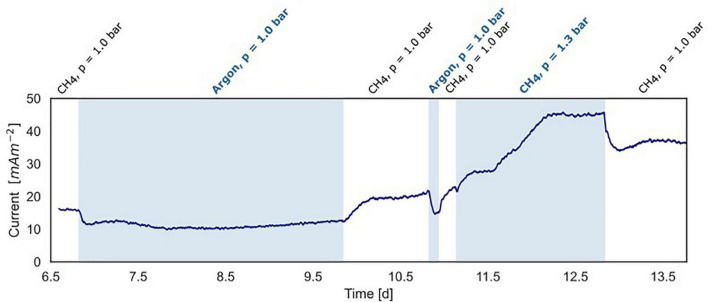
Current production under varying methane concentrations in the BES headspace (experiment B). White areas show standard conditions at atmospheric pressure with CH_4_/CO_2_ (95%/5%) and N_2_ flushed through the anode chamber. The blue areas show non-standard conditions with-in the first and second part, argon/CO_2_ flushed through the system at atmospheric pressure, replacing CH_4_/CO_2_. In the third part, the gas in- and outflow was interrupted and 30 ml CH_4_ was added to the anode with a CH_4_/CO_2_ (95%/5%) headspace resulting in an overpressure of 0.3 bar.

From inoculum to biofilm, the relative abundance of archaea increased from 55 to 63% and the relative abundance of bacteria decreased from 44 to 36%. At lower taxonomic levels, the changes in abundance were minor. Within 8 days, no large differences were expected as “*Ca.* Methanoperedens” has a relatively high doubling time of weeks to months (Raghoebarsing et al., 2006; Vaksmaa et al., 2017). The data indicate that “*Ca.* Methanoperedens” was dominant in both the inoculum and the biofilm in the BES, and no other fast-growing community members emerged at the bioanode over this time period. “*Ca.* Methanoperedens” had a relative abundance of 47% at the bioanode. All other community members did not appear above 2% abundance. The well-known electroactive family of *Geobacteraceae* had a low relative abundance of 0.6% in the bioreactor and was 0.5% in the biofilm. Also, *Methanosarcinaceae* was found to be the part of the microbial community in the biofilm with a low relative abundance of 0.6%.

### The Microbial Community Dominated by “*Ca.* Methanoperedens” Is Electroactive

In this work, we investigated the ability of a “*Ca*. Methanoperedens” dominated culture to perform EET in a BES. We used a “*Ca.* Methanoperedens” dominated bioreactor culture that performed AOM with nitrate as the electron acceptor as the inoculum. We performed three independent BES experiments (A, B, and C) in batch mode.

At inoculation, the current density was about 0 mA m**^–^**^2^ for all three experiments using a poised potential of 0.0 V vs. SHE (Figure 2). Shortly after inoculation, the current density started to increase quickly, which reaches a maximum of 274 mA m**^–^**^2^ (493 μA) after 19.4 h for experiment A, 132 mA m**^–^**^2^ (238 μA) after 31.9 h for experiment B, and 99.0 mA m**^–^**^2^ (178 μA) after 27.7 h for experiment C. After the peak, the current decreased and stabilized at 8.0 mA m**^–^**^2^ (14 μA) (experiment A), 15 mA m**^–^**^2^ (27 μA) (experiment B), and 39 mA m**^–^**^2^ (70.0 μA) (experiment C). Controls, either without biomass or with autoclaved biomass, were used to exclude the possibility of abiotic current production; no current was produced in either control (Supplementary Figure 3). Furthermore, we ensured that no leftover nitrate was present in the inoculum to function as the electron acceptor. Combined these 3 experiments showed that the microbial community was immediately electroactive.

With “*Ca.* Methanoperedens” as the most dominant microorganism in the inoculum, it was investigated whether the produced electrical current was dependent on methane availability in three individual batch experiments described in sections “CH_4_ Conversion Into CO_2_ Co-occurs With Current Production, and Part of the Current Produced in the Bioelectrochemical System Is Dependent on Methane Availability. Polarization Curves Show a Limitation for Methane Dependent Current”

### CH_4_ Conversion Into CO_2_ Co-occurs With Current Production

In experiment A (Figure 2A), the relation between current production and methane consumption was quantified by measuring the consumption of CH_4_ in the anode chamber (Figure 4, triangles and circles, Figure 2A, gray part). Methane was consumed with concomitant current production, and this corresponded to a Coulombic efficiency of 17.3%. Coulombic efficiency was calculated by dividing the amount of electrons harbored by the oxidized methane (8 electrons per methane molecule) divided by the amount of current in electron equivalents. The enrichment of ^13^CO_2_ (% ^13^CO_2_/total CO_2_) was experimentally validated with an increase from 1.1 to 1.4%, which demonstrated methanotroph activity (Figure 4, crosses). A maximum current density of 274 mA m**^–^**^2^ was reached during this experiment (Figure 2A). The methane consumption rate in batch incubations with nitrate as the electron acceptor was 24.2 ± 5.9 nmol CH_4_ mgDW biomass**^–^**^1^ h**^–^**^1^, while in the BES, a rate of 6.80 ± 0.81 nmol CH_4_ mgDW biomass h**^–^**^1^ was observed (Supplementary Figure 2).

### Part of the Current Produced in the Bioelectrochemical System Is Dependent on Methane Availability

In experiment B (Figure 2B), at a stable current of 15 mA m**^–^**^2^ reached after 6.8 days, the supplied CH_4_/CO_2_ gas was replaced by Argon/CO_2_ with no other changes in the system (pressure, CO_2,_ and N_2_ availability). Upon the removal of methane from the gas inflow, the current decreased from 16 to 10 mA m**^–^**^2^ after which it stabilized (Figure 5). We estimate that this difference of 38% represents the current that is dependent on methane oxidation, whereas 62% of current generation may have been caused by other processes, for example, heterotrophic dissimilation or the breakdown of storage compounds.

After the stabilization of the current at 10 mA m**^–^**^2^, methane was added again which resulted in a subsequent increase of the current to the previous level of 15 mA m**^–^**^2^ (Figure 5). A similar methane removal/re-addition experiment was repeated after 10.8 days for a shorter period and gave the same response. To further study the dependency of current generation on methane supply, the anode chamber was closed and the gas inflow was stopped at a current of 22 mA m**^–^**^2^ after 11.1 days. Subsequently, 30 ml of methane was added to the anolyte, which results in an overpressure of 0.3 bar. The current increased upon methane addition and stabilized at 45 mA m**^–^**^2^ (Figures 2B, 5), which indicates that methane is most likely the limiting component in the process. After depressurizing the anode chamber and resuming the gas inflow at atmospheric pressure, the current decreased again to a value of 35 mA m**^–^**^2^. These two experiments demonstrate that part of the current is directly dependent on the methane availability.

### Polarization Curves Show a Limitation for Methane Dependent Current

**FIGURE 6:**
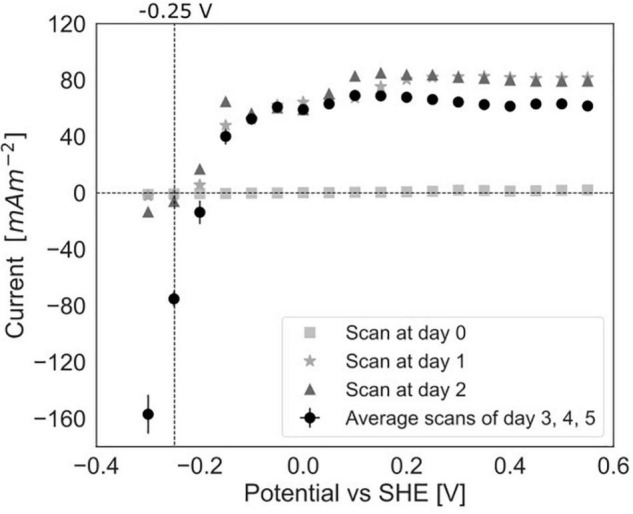
Polarization curves in experiment C at day 0, after 1 day, after 2 days of incubation in the BES and an average of the curves after 3, 4, and 5 days (error bars show standard deviation, *n* = 3).

To study the dependency of methane oxidation on the potential, polarization curves were recorded over a range of −0.35 to 0.50 V vs. SHE, which started right after inoculation and was continued daily until 5 days after inoculation for experiment C (Figures 2C, 6). At t0, no current was produced for the tested potential range, which indicates that no electrochemical methane oxidation was possible at the investigated potentials. No biofilm had been formed yet at t0. From day 1 onward, the current was produced, and a biofilm was visible on the electrode, which suggests the formation of an electroactive biofilm. After day 1, the current was stable at potentials higher than 0 V, which demonstrates that an increase in the anode potential could not boost the current production anymore. This implies a limitation that is either related to substrate availability or to microbial activity. The microorganisms started oxidizing the substrate and transferring electrons to the electrode (current changed from negative to positive) at a potential of approximately −0.19 V vs. SHE at days 3, 4, and 5. This redox potential is close to the theoretical potential of the redox couple CH_4_/CO_2_ calculated for the conditions in the BES being −0.249 V vs. SHE (with conditions: pH 7.25, 86.4% of the gas phase CH_4_, 4.55% of the gas phase CO_2_).

### Three Different Methanoperedens Metagenome-Assembled Genomes Were Found in the Biofilm

Genomes were assembled from the metagenome to analyze the microbial community in more detail and to obtain more insight into the potential EET mechanisms. Taxonomy was assigned to the MAGs using GTDB-tk (Chaumeil et al., 2020). Three different “*Ca*. Methanoperedens” MAGs were assembled with a completeness of 93% for Methanoperedens #1 and 72% and 59% for #2 and #3, respectively. Methanoperedens #1 dominated both the inoculum and the biofilm at the anode, with 33% of the reads mapped to this MAG in the inoculum compared to 41% in the biofilm, while #2 and #3 had a relative abundance of 3.3 and 1.9% in the inoculum compared to 2.2 and 2.7% in the biofilm. All three MAGs were most closely related to “*Ca.* Methanoperedens” BLZ2 (Berger et al., 2017). Based on the ANI assessed by JSpeciesWS, Methanoperedens #1 was 87% identical to BLZ2, #2 was 98%, and #3 was 92% identical to strain BLZ2. Based on the digital DNA-DNA hybridization [identities/high-scoring segment pairs (HSP) length], Methanoperedens #1 was 36.4% similar to BLZ2, #2 was 88.1% similar to BLZ2, and #3 was 52.3% similar to BLZ2 (Meier-Kolthoff et al., 2013).

### Cytochromes Encoded in the Genome of Methanoperedens Metagenome-Assembled Genome #1 May Be Involved in the Extracellular Electron Transfer Mechanism

**FIGURE 7:**
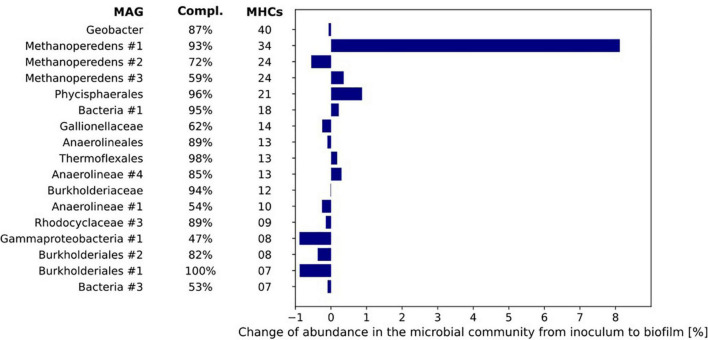
The number of multiheme cytochromes (MHCs) harbored by the MAGs of the microorganisms with most MHCs (threshold of ≥ 4 MHC). The MAGs are sorted from top to bottom by their number of *c*-type cytochromes. The bar chart represents the differences in relative abundance of the MAGs in inoculum and biofilm based on coverage of the respective MAGs under both conditions.

As MHCs are mostly found in microorganisms performing EET such as *Geobacter*, *Shewanella*, and anammox bacteria (Logan, 2009; Shaw et al., 2020), the MAGs were mined for these proteins. Figure 7 provides an overview of the MAGs that contained seven or more proteins with more than three C-X-X-C-H heme-binding motifs (Supplementary Tables 2–4).

The difference in relative abundance between inoculum and biofilm was highest for MAG Methanoperedens #1 where the relative abundance in the biofilm was 8.1% higher than in the inoculum. Further, it encoded no less than 34 MHCs. The *Geobacter* MAG contained 40 putative MHCs but its relative abundance was the same in both inoculum and biofilm (0.6–0.7%, Figure 7).

As the primary candidate for EET to the electrode producing the methane-dependent current, the genome of “*Ca.* Methanoperedens” was further investigated. Thirty-four MHCs were identified in the MAG of Methanoperedens #1 with 3–38 heme-binding sites, seven of which were predicted to be anchored to the cytoplasmic membrane (Supplementary Table 4). No mono- and di-heme c-type cytochromes were identified. Consistent with the previously described “*Ca*. Methanoperedens” strains, “*Ca.* Methanoperedens” MAG #1 encoded two MHCs with S-layer domains (METP1_00516, METP1_03209) (McGlynn et al., 2015; Cai et al., 2018; Leu et al., 2020a). They comprised 23 and 5 CXXCH motifs, respectively. Although metatranscriptomics are not directly related to the measured electroactivity results, the possibility of the involvement of some of the MHCs was indicated by metatranscriptome data from the bioreactor. These data show several of these MHCs to be already highly expressed in the bioreactor compared to the expression of several genes of the reverse methanogenesis pathway and gene-encoding enzymes involved in nitrate reduction (Supplementary Tables 4, 5). The increase in current production immediately after inoculation of the BESs indicates that (part of) the proteins involved in EET were already present in the bioreactor.

Other redox mediators that are often part of the EET mechanism are electron shuttles such as flavins and phenazines (Kracke et al., 2015; Liu et al., 2018). These function as electron carriers that can mediate electron transfer from the cytoplasmic membrane to an external electron acceptor. The Methanoperedens #1 MAG was mined for gene-encoding proteins involved in the biosynthesis pathways of phenazine and flavins. The biosynthesis pathway of phenazine (including proteins: PhzA, PhzB, PhzC, PhzM, and PhzH), an electron shuttle reported to be used by other microorganisms, was not found in MAG Methanoperedens #1 (Mavrodi et al., 2001; Yong et al., 2014). The biosynthesis pathway of flavins, an electron shuttle used by *Shewanella oneidensis, Bacillus subtilis*, and *Escherichia coli*, was encoded in the Methanoperedens #1 MAG (Brutinel and Gralnick, 2012; Yang et al., 2015): RibA, RibB, RibE, and RibH were encoded in the genome (METP1_00229, METP1_01194, METP_00154, and METP1_03625). These flavins, however, are also used as co-factor for many enzymes in anaerobes such as “*Ca.* Methanoperedens” and would thus require more detailed investigation for their involvement in EET.

## Discussion

### A Newly Found “*Ca.* Methanoperedens” Strain Is the Most Dominant Microorganism in the Biofilm

In this study, we showed current production in a BES poised at 0.0 V vs. SHE that is partly methane dependent. Metagenomic data showed that after a period of 8 days, the anaerobic methanotroph “*Ca.* Methanoperedens” was still the most dominant microorganism in the culture while no other (electroactive) microorganisms emerged at the anode. The dominant “*Ca.* Methanoperedens” strain found in these experiments has not been previously described. It is most similar (87% ANI, 36.4% DDH) to the previously described BLZ2 strain (Berger et al., 2017).

### As Sole Methanotroph, “*Ca.* Methanoperedens,” Produces Methane-Dependent Current in the Bioelectrochemical System

We experimentally validated the oxidation of methane to CO_2_ while current was produced and further demonstrated that “*Ca.* Methanoperedens” was responsible for (part of) the electrical current produced. The methane consumption rate in the BES was fourfold lower compared to nitrate-dependent methane conversion by the same culture, which was previously also observed when iron citrate or ferrihydrite was used as the electron acceptors (Ettwig et al., 2016). In the MFC inoculated with a “*Ca.* Methanoperedens” enrichment by Ding et al. (2017), a maximum current density of 21 mA m**^–^**^2^ was produced (calculated from the applied resistance of 1,000 Ohm, the surface area of 12 cm**^–^**^2^ and the maximum electricity production of 25 mV). This shows the relatively high activity of the microbial community at the anode in the present study. Experiment B, in which an overpressure of methane was added, showed that methane is limiting under atmospheric pressure. Polarization curves demonstrated that an increase in potential did not increase current production by the community. A methane limitation under atmospheric pressure used for the polarization curves could possibly explain this finding. Over time, the potential at which the microorganisms donated electrons to the anode (−0.19 V vs. SHE) became close to the redox potential of the CH_4_/CO_2_ couple under actual conditions (−0.249 V vs. SHE), which supports our finding that methane is the driving force for current production. At the voltage where the current density is zero, no reaction occurs and the measured potential should be equal to the theoretical potential. In reality, this open circuit potential is slightly different from the theoretical value as the conditions at the electrode surface are not exactly the same as in the anolyte. If the potential exceeds (is more positive than) the potential of the redox couple, the Gibb’s free energy change of the reaction becomes more negative and current can be produced (Ter Heijne et al., 2019). The depletion of methane from the medium showed that 38% of the generated current was directly dependent on the methane supply. Besides the anaerobic methanotroph “*Ca*. Methanoperedens,” a methanogen from the family *Methanosarcinaceae* was found in the biofilm at the electrode. Surprisingly, a *Methanosarcina* sp. has been previously found to be capable of EET in a BES at an anode poised at 300 mV (Yu et al., 2021). In our experiments, the relative abundance of this microorganism in the biofilm at the electrode was low with 0.6%. “*Ca*. Methanoperedens,” the sole methanotroph in the community, was most likely responsible for this current generation.

### No Fast-Growing Electroactive Microorganisms Emerged at the Electrode

We did not study the remainder of the generated current, possible explanations could be that the current was produced by other community members *via* heterotrophic conversion of dead biomass, or “*Ca*. Methanoperedens” contributed to the current generation *via* the use of internal storage compounds such as polyhydroxyalkanoate (PHA) (Cai et al., 2019). The candidates for current production by heterotrophic processes are the microorganisms encoding a large number of MHCs (Figure 7), such as *Geobacter*, Phycisphaerales, and an unclassified bacterium. These methane-independent processes, however, did not lead to a higher relative abundance of any bacterium in the biofilm compared to the inoculum within the operation time of 8 days used in the BES. “*Ca.* Methanoperedens” was the most dominant microorganism in both the inoculum and the biofilm with relative abundances of 42% in the inoculum to 47% in the biofilm. This, together with the methane-dependent current, suggests that we may be able to further enrich these microorganisms using such a BES set-up to achieve the long-term goal of a first axenic ANME culture. Longer experiments and following the amount of biomass and “*Ca.* Methanoperedens” cell numbers are needed in future experiments. The 8 days in which the BES was operated is probably too short to see a large change in the microbial community composition. Nevertheless, it provides insights into the activity of the microorganisms. While anaerobic methane oxidation is associated with slow growth rates (Lu et al., 2019), heterotrophic EET, for example by *Geobacter* is known to support fast growth rates (Marsili et al., 2010). As we did not observe any increase in relative abundance from inoculum to biofilm of heterotrophic bacteria, which includes *Geobacter* at the bioanode, we assume that heterotrophic EET as the driving process at the bioanode is not very likely.

### The Presence of Storage Polymers May Be Responsible for a Peak That Emerges After Electron Acceptor Limiting Conditions

At the start of all three batch experiments, a sharp peak in current production was visible which was not observed when autoclaved biomass was used for the experiment. We hypothesize that this peak may be related to the fast consumption of accumulated storage polymers, which were previously detected in “*Ca.* Methanoperedens” (Cai et al., 2019). The enrichment culture that was used as inoculum was continuously exposed to an excess of methane over nitrate supply, that is, nitrate-limiting conditions, which explains the production of storage polymers in this culture. This is confirmed by the metatranscriptomics data from the enrichment culture used for inoculation which showed high expression of several genes involved in PHA metabolism (Supplementary Table 5). In an experiment with excess electron donor (methane) and interruption of the applied potential (open circuit), we observed similar peaks (Supplementary Figure 4) that showed a direct relationship between the length of the open circuit and the peak size, which suggests that indeed storage polymers might have been produced by the microbial community during the open circuit and used as soon as the potential was reapplied.

### Phycisphaerales and an Unknown Bacterium May Have Replaced Commonly Found Nitrite Scavengers “*Ca.* Methylomirabilis” and “*Ca.* Kuenenia” in the Inoculum Enrichment Culture

Surprisingly, in our inoculum enrichment culture community, “*Ca.* Methylomirabilis” and “*Ca.* Kuenenia” were absent. These microorganisms are commonly found in “*Ca.* Methanoperedens” enrichments, scavenging toxic nitrite, produced from the reduction of nitrate by the methanotroph. Alternatively, both the Phycisphaerales MAG and the MAG from the unclassified bacterium were found to encode enzymes involved in nitrite reduction (PHYC_00865, PHYC_00866, BAC1_02213, BAC1_02220), which suggests that these bacteria had taken over the detoxification of nitrite in the inoculum enrichment culture in the absence of “*Ca*. Methylomirabilis” and anammox bacteria.

### Several Multiheme *c*-Type Cytochromes May Be Involved in Extracellular Electron Transfer

It has been hypothesized that MHCs may be involved in the EET mechanism of ANMEs as their genomes have been found to encode a large number of MHCs (Kletzin et al., 2015; McGlynn et al., 2015; Leu et al., 2020b), which are typically reported in electroactive microorganisms as part of their EET pathway (Logan, 2009). In cultures fed with ferrihydrite and birnessite and dominated by “*Ca.* Methanoperedens,” transcriptomic data showed the expression of several MHCs (Cai et al., 2018; Leu et al., 2020a). Similarly, Methanoperedens #1 MAG encoded a large number of MHCs. Unfortunately, the RNA quantity obtained from the electrode biomass was insufficient to perform RNA metatranscriptome sequencing so only metatranscriptome data from the enrichment culture were available. Immediately after the inoculation of the BES, current was produced by our microbial community (Figure 2). This indicates that (part of) the proteins involved in EET may have already been expressed at the onset of the experiment. For the metatranscriptome data of the inoculum, we compared the expression of MHCs harbored by “*Ca*. Methanoperedens” to the expression of regular reverse methanogenesis genes such as *mcr, mer, mtd, mch*, and *nar*. The nitrate reductase *narGH* is used by “*Ca.* Methanoperedens” to convert nitrate to nitrite. We identified twenty MHCs with an expression higher than *nar* of which four were predicted to be anchored to the cytoplasmic membrane (Supplementary Tables 4, 5). One MHC even had an expression comparable to *mcrA* (Supplementary Tables 4, 5). These data cannot be directly linked to the electroactive culture but provide a hint toward the possible involvement of these MHCs in EET by “*Ca*. Methanoperedens.” These highly expressed MHCs will be subject of future studies on the possible mechanism of EET by “*Ca.* Methanoperedens.”

### Conclusion

The BES used in this study provides a suitable environment for “*Ca.* Methanoperedens” to produce methane-dependent current. The fact that “*Ca.* Methanoperedens” was the most dominant microorganism in the inoculum and in the biofilm, while no (fast growing) bacteria emerge at the biofilm and methane-dependent current occurs suggests that this methanotroph is capable of EET, which produces electrical current at the anode of a BES. Our work describes a system in which ANME-mediated EET can be investigated under controlled laboratory conditions, opening new research opportunities for mechanistic studies and possibly the generation of axenic ANME cultures.

## Data Availability Statement

The datasets presented in this study can be found in online repositories. The names of the repository/repositories and accession number(s) can be found in the article/Supplementary Material.

## Author Contributions

HO, AH, TS, and CW planned the research. HO and SB performed the research. HO and TB analyzed data. CW and MJ obtained funding for the research project. HO wrote the manuscript with contributions from TB, SB, MJ, TS, AH, and CW. All authors contributed to the article and approved the submitted version.

## Conflict of Interest

The authors declare that the research was conducted in the absence of any commercial or financial relationships that could be construed as a potential conflict of interest.

## Publisher’s Note

All claims expressed in this article are solely those of the authors and do not necessarily represent those of their affiliated organizations, or those of the publisher, the editors and the reviewers. Any product that may be evaluated in this article, or claim that may be made by its manufacturer, is not guaranteed or endorsed by the publisher.

## Footnotes

1
http://jspecies.ribohost.com/jspeciesws/

2
https://www.ncbi.nlm.nih.gov/Structure/cdd/wrpsb.cgi
