# Supplementary material for: Methane-Dependent Extracellular Electron Transfer at the Bioanode by the Anaerobic Archaeal Methanotroph “Candidatus Methanoperedens”
Source: Front Microbiol. 2022 Apr 12;13:820989. doi: 10.3389/fmicb.2022.820989 (PMC9039326; doi:10.3389/fmicb.2022.820989)
Supplement: Supplementary file 1 [file Data_Sheet_1.pdf]

Methane-dependent extracellular electron transfer at the bioanode by the anaerobic archaeal methanotroph '*Candidatus* Methanoperedens'

Heleen T. Ouboter[a], Tom Berben[a], Stefanie Berger[a], Mike S.M. Jetten[a], Tom Sleutels[b], Annemiek Ter Heijne[c], Cornelia U. Welte[a]

Supplementary Material

Supplementary Methods

Calculation of actual redox potential in the BES

Due to the non-standard conditions in the BES, we have calculated the resulting redox potential of the CH_4/CO_2 redox couple according to the Nernst equation and the following parameters:

$$E' = E0' + 2.3 \times \frac{R \times T}{n \times F} \times log10 \ \frac{[oxidized]}{[reduced]}$$

E' = adjusted redox potential under biological conditions = -0.249 V

E0' = standard redox potential under biological conditions = -0.24 V

R = gas constant, 8.314 J/(mol*K)

T = temperature in Kelvin, 296 K

n = number of transferred electrons, 8

F = Faraday's constant, 96500 J/(mol * V)

[oxidized], CO_2 partial pressure in bar, 0.045 bar (measured with the gas flow)

[reduced], CH_4 partial pressure in bar, 0.86 bar (measured with the gas flow)

Experiment	Tests performed
A	$^{13}CH_4$ labelling experiment + metagenome data of inoculum and biofilm and metatranscriptome data of the inoculum
B	Methane replacement for argon and vice versa and creating an overpressure of methane to test methane dependent current
C	Polarisation scans

Supplementary Figure 1: Overview of the performed experiments.

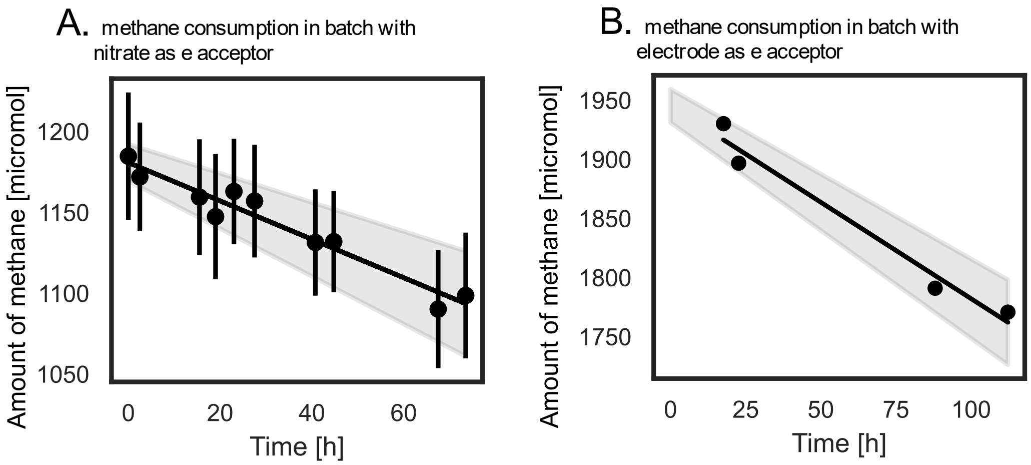

Supplementary Figure 2: Methane consumption in a batch with nitrate as electron acceptor (A) and with the electrode as electron acceptor (B). For both experiments, a linear regression was used to calculate the consumption rate, the standard deviation of this regression is shown in grey. For the nitrate experiment, the average results are shown of three batch experiments with standard deviations. Dry weight of the biomass used in the experiment was for A) 49 mg, and for B) 240 mg. The specific activity was for A) -24.2 ± 5.9 nmol CH_4 mgDW biomass^{-1} h^{-1} and for B) -6.80 ± 0.81 nmol CH_4 mgDW biomass h^{-1}

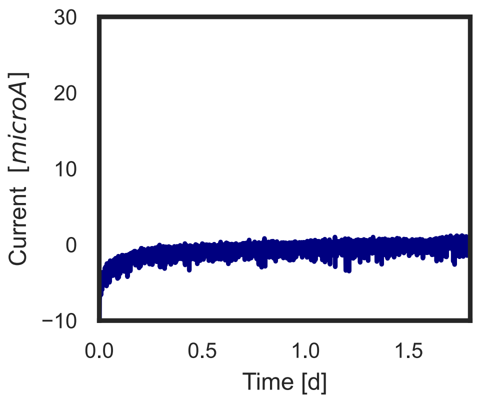

Supplementary Figure 3: Current production in a BES inoculated with autoclaved biomass (negative control)

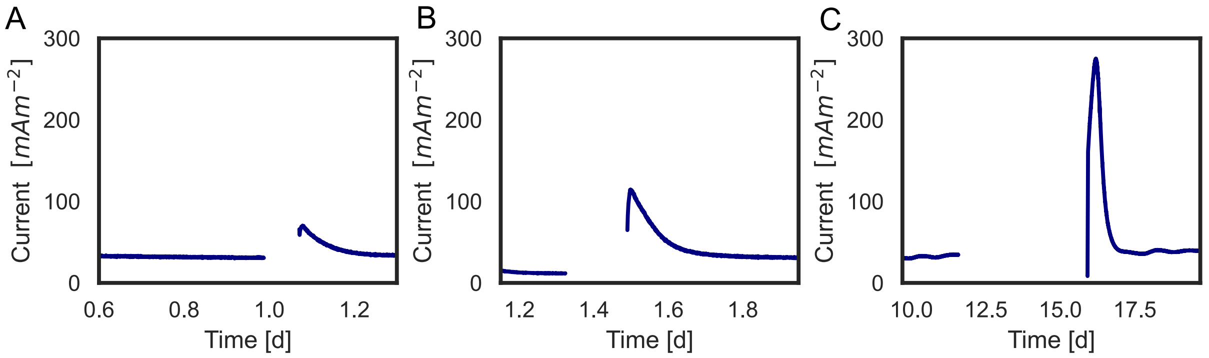

Supplementary Figure 4: Current production in a BES in which chronoamperiometry was interrupted by open circuit potentiometry. For all three experiments, current before and after the interruption is shown, with no current shown during OCP. A) Interruption of chronoamperiometry by 2 h, B) Interruption of chronoamperiometry by 4 h, C) Interruption of chronoamperiometry by 4 days.

Supplementary Table 1: Relative abundance of the microorganisms assessed by assigning metagenomic sequencing reads at the nucleotide level

Supplementary Table 2: Overview of the metagenome-assembled genomes (MAGs)

Supplementary Table 3: Overview of the multi-heme cytochromes (MHCs)

Supplementary Table 4: MHCs MAG Methanoperedens #1 with transcriptome data

Supplementary Table 5: RNA seq data of reference genes involved in reverse methanogenesis + genes related to PHA metabolism
